# Did the Number of Older People Requiring Long-Term Care and Expenditure Increase after the 2011 Great East Japan Earthquake? Analysis of Changes over Six Years

**DOI:** 10.3390/ijerph17051621

**Published:** 2020-03-03

**Authors:** Yusuke Inoue, Seungwon Jeong

**Affiliations:** 1 Department of Welfare Systems and Health Science, Okayama Prefectural University, Soja, Okayama 719-1197, Japan; 2 Department of Community Welfare, Niimi University, Niimi, Okayama 718-8585, Japan; k-jeong@niimi-u.ac.jp

**Keywords:** the 2011 Great East Japan Earthquake, long-term care certification rate, expenditure of long-term care services

## Abstract

On 11 March 2011, the great earthquake hit Japan, resulting in 15,895 deaths, 6156 serious injuries, and 2539 missing persons. This event affected the health and lives of older residents, and reports showed an increase in the number of people eligible for long-term care afterward. In this study, among the places affected by the 2011 Great East Japan Earthquake and tsunami, we focused on 15 municipalities, including designated municipalities based on the Special Act on Nuclear Evacuation in Fukushima Prefecture, and aimed to clarify the medium-term effects (six years post-disaster) on the long-term care certification rate and expenditure for provision of services. We used the Ministry of Health, Labour, and Welfare Monthly Status Report on Long-Term Care Insurance and the Ministry of Internal Affairs and Communications Population Register for 2011, 2014, and 2017. In 2011, we found no intergroup differences among the 15 Fukushima municipalities and other municipalities in either the long-term care certification rate or the per-person expenditure for use of services. In 2014, after the earthquake, the long-term care certification rate was 5.4% higher in the 15 Fukushima municipalities than in other municipalities for those aged 75 years or older. The rate of 2014–2017 has not increased significantly, partly because of stability after the disaster and change in the population structure. Nevertheless, the long-term care certification rate in the 15 Fukushima municipalities is higher than that of the other two groups even after six years since the earthquake. Similarly, the per-person expenditure for use of services for one month was 11,800 yen higher in the 15 Fukushima municipalities than in other municipalities in 2014, and this trend continued into 2017. Strong, ongoing governmental support is needed, especially for those aged 75 or older, following a disaster.

## 1. Introduction

Disasters cause numerous casualties [1,2,3] and negatively impact the survivors [4,5]. Previous studies indicated that besides direct injuries in the disaster, there is an increase of acute stress, depression, and heart disease [6,7,8,9]. Disasters particularly affect vulnerable older people [10,11]. On March 2011, the great earthquake hit Japan, resulting in 15,895 deaths, 6156 serious injuries, and 2539 missing persons. The Fukushima power plant incident and destruction of homes caused by the resulting tsunami left many people living in evacuation centers. These events significantly affected the health and lives of older residents [12], and reports showed an increase in the number of people eligible for long-term care [13].

Areas in the Fukushima, Iwate, and Miyagi prefectures that were devastated by the tsunami had a higher long-term care certification rate from 2011 to 2014 compared to other areas [14,15]. In the 190 municipalities to which the Disaster Relief Act applied, the number of residents aged 75 years and older who required medium-term care increased after the earthquake (2009 vs. 2011) [16]. We examined the change in the number of people certified for long-term care over the three-year period from 2011 to 2014 in 15 municipalities in Fukushima, including designated municipalities based on the Special Act on Nuclear Evacuation, (Iwaki city, Soma city, Tamura city, Minami-Soma city, Kawamata town, Hirono town, Naraha town, Tomioka town, Kawauchi village, Okuma town, Futaba town, Namie town, Katsurao village, Shinchi town, and Iitate village) that were particularly destroyed by the tsunami, and found that the number of people requiring minor or medium care increased [17].

Studies to date have examined changes for about three years after the earthquake, but the impact of the earthquake may not have been short-lived and may have affected the health and behavior of residents over the medium to long term. Therefore, studying the number of people certified for long-term care and other factors from a medium- to long-term perspective would be useful.

In this study, among the places affected by the 2011 Great East Japan Earthquake and tsunami, we focused on 15 coastal municipalities in Fukushima Prefecture that were particularly devastated by the tsunami, and aimed to clarify the medium-term effects (six years post-disaster) of a major disaster, a tsunami, on the long-term care certification rate and expenditure for provision of services.

## 2. Method

### 2.1. Data

In this study, we used the Ministry of Health, Labour, and Welfare Monthly Status Report on Long-Term Care Insurance for January 2011, 2014, and 2017, and the Ministry of Internal Affairs and Communications Population Register for 2011, 2014, and 2017. The Monthly Status Report on Long-term Care Insurance data and Population Register data for 2011, 2014, and 2017 were combined. The number of people certified for long-term care and the number of people receiving services were obtained from the Monthly Status Report on Long-Term Care Insurance. The per-person expenditure for use of services was calculated by dividing the total cost of each service (home-based services, community, facility) by the total number of users. Long-term care certification rate was calculated by dividing the number of people certified for long-term care by the population aged 65 and over. The ratio of people aged 75 or older was calculated by dividing the population 75 years or older in the population by the population 65 years or older. The population data was obtained from the Ministry of Internal Affairs and Communications.

The municipalities were divided into three groups for inter-regional comparison: (1) 15 municipalities in Fukushima that were severely impacted by the tsunami (“15 Fukushima municipalities”), (2) municipalities in Fukushima other than those mentioned in (1) and municipalities in the Miyagi and Iwate prefectures that neighbor Fukushima (“nearby municipalities”), and (3) municipalities in other prefectures (“other municipalities”).

### 2.2. Long-Term Care Insurance Program in Japan

Medical insurance and long-term insurance are operated by the national insurance program in Japan. Medical insurance was founded in 1961. Additionally, Japan has provided a long-term care insurance program since 2000 (social insurance system) for the increasing older population. Long-term care insurance is broadly divided into institutional services and home-based services. Receiving long-term care services requires long-term care certification. The long-term care certification process begins with an application by the individual, followed by examination of their mental and physical condition by certification screening personnel and a diagnosis from their primary physician. Based on those results, the municipality that is their insurer conducts a screening by its long-term care certification screening committee and makes the final decision on the level of care required. Aside from noneligibility, seven levels of care are available: levels 1 and 2 (support needs) and levels 3 to 7 (long-term care needs) [18]. As of the end of 2017, roughly 18% (6.42 million people) of the 34.85 million residents aged 65 years or older have been certified for long-term care need. About 86% of long-term care recipients are aged 75 years or older. With long-term care certification, the service can be paid for by insurance. Service user copayments at the rate of 10%–20% depending on their income are collected as part of the multitiered funding mechanism for the long-term care insurance program.

## 3. Analysis Method

### 3.1. Descriptive Statistics

To determine the status before the earthquake, the number of people eligible by care level and number of people receiving services in January 2011 were examined for the (1) 15 Fukushima municipalities, (2) nearby municipalities, and (3) other municipalities, and the rate of increase from 2011 to 2017 was examined. Care level was divided into minor (support need levels 1 or 2, long-term care need level 3), medium (long-term care levels 4 or 5), and major (long-term care levels 6 or 7), and each group was then divided into the ratio of those aged 65 years or older (population aging rate) and the ratio of those aged 75 years or older.

### 3.2. Analysis of Repeated Measures ANOVA

A repeated measures analysis of variance (RM-ANOVA) was performed to test for differences among the three groups, considering between the effects of time (2011, 2014, 2017) and the effects of earthquake and tsunami. The items for analysis included the long-term care certification rate and per-person expenditure for use of long-term care insurance services for 2011, 2014, and 2017. As the long-term care certification rate generally increases with increasing age, the long-term care certification rate was higher in municipalities with a larger ratio of individuals aged 75 years or older in the 65 years and older population. To eliminate the effect of that factor, the ratio of those aged 75 years or older was used as a covariate in the analysis. Mauchly’s Test of Sphericity indicated that the assumption of sphericity had been violated, and therefore, a Greenhouse–Geisser correction was used. SPSS Statistics for Windows, Ver. 24 (IBM, Armonk, NY, USA) was used for the analysis.

## 4. Results

### 4.1. Numbers of People Eligible for Long-Term Care and People Receiving Services

The population aging rate before the earthquake (January 2011) was lower in the 15 Fukushima municipalities (26.5%) than in the nearby municipalities (28.1%) and other municipalities (27.4%). A similar trend was observed in 2014 and 2017. The ratio of old-old in the population in 2011 was the highest in the 15 Fukushima municipalities (57.6%). The ratio was next highest in the nearby municipalities (56.2%), followed by the other municipalities (52.5%). In 2017, the ratio was 53.5% in the 15 Fukushima municipalities, 54.2% in the nearby municipalities, and 51.8% in the other municipalities (Table 1).

The number of people certified for long-term care increased from 2011 to 2014 more in the 15 Fukushima municipalities than in the nearby municipalities and other municipalities among both the young-old (those aged 65–74 years) and the old-old (those aged 75 years or older) for those with minor or medium need. The ratio of those with minor needs increased more among the 15 Fukushima municipalities (increase of 25.4% for the young-old and 31.7% for the old-old) than the nearby municipalities (increase of 13.2% for the young-old and 21.8% for the old-old) and other municipalities (increase of 19.0% for the young-old and 22.6% for the old-old). The ratio of those with medium need also increased more among the 15 Fukushima municipalities (increase of 12.5% for the young-old and 30.0% for the old-old) than the nearby municipalities (increase of 3.7% for the young-old and 16.5% for the old-old) and other municipalities (increase of 6.1% for the young-old and 14.6% for the old-old). However, the ratio of those with major need for the entire 65+ population increased the least in the 15 Fukushima municipalities (3.6% increase) compared to the nearby municipalities (7.5%) and other municipalities (8.5%), and actually decreased by 10.7% among the young-old in the 15 Fukushima municipalities (Table 1).

From 2014 to 2017, the increase in the ratio was about the same or slightly lower in the 15 Fukushima municipalities for those with minor (increase of 6.8% for the young-old and 9.7% for the old-old) and medium needs (increase of 8.6% for the young-old and 8.9% for the old-old) compared to the nearby and other municipalities. For the entire 65+ population, the ratio of those with major need increased 3.2% in the nearby municipalities and 4.1% in the other municipalities, and decreased 2.1% in the 15 Fukushima municipalities (Table 1).

The number of those receiving services increased more from 2011 to 2014 in the 15 Fukushima municipalities for those with minor need (24.6% increase) and medium need (25.0%) compared with the nearby municipalities (18.5% increase for those with minor need and 17.4% increase for those with medium need) and other municipalities (21.3% increase for those with minor need and 15.8% increase for those with medium need). Although the increase slowed leading up to 2017, the ratio was still higher in the 15 Fukushima municipalities for those with minor (19.5% increase) and medium needs (22.6%) compared to the nearby municipalities (14.8% increase for those with minor need and 18.4% increase for those with medium need) and other municipalities (16.1% increase for those with minor need and 20.9% increase for those with medium need). The ratio of those with major need increased less in 2014 in the 15 Fukushima municipalities (2.7%) than in the nearby municipalities (9.6%) and other municipalities (12.9%). A similar trend was seen in 2017 (Table 1).

### 4.2. Results of Repeated Measures ANOVA

We examined intergroup differences among the 15 Fukushima municipalities, the nearby municipalities, and the other municipalities in the long-term care certification rate and per-person expenditure for use of long-term care insurance services (Table 2).

The long-term care certification rate of the people aged 75 years or older was lower in the 15 Fukushima municipalities in 2011 (25.8%) compared to the other two groups (26.1% for nearby municipalities, 28.2% for other municipalities). In contrast, it was significantly higher (*p* < 0.001) in the 15 Fukushima municipalities than in the other two groups in both 2014 (34.7% for the 15 Fukushima municipalities, 29.3% for the nearby municipalities, 30.3% for the other municipalities) and 2017 (36.5% for the 15 Fukushima municipalities, 30.4% for the nearby municipalities, 30.6% for the other municipalities).

Per-person expenditure for use of services over one month did not differ among the three groups in 2011, but was higher in the 15 Fukushima municipalities (155,900 yen) compared to the nearby municipalities (144,100 yen) and other municipalities (145,500 yen) in 2014. Although overall expenditure was lower in 2017 than in 2014 in all three groups, it was higher in the 15 Fukushima municipalities (147,000 yen) compared to the other two groups (138,900 yen and 134,900 yen, respectively) in 2017.

Moreover, a post hoc test was conducted after we completed an RM-ANOVA in order to determine which groups differed from each other. On the long-term care certification rate of all and per-person expenditure for use of services, there was a significant effect of time (2011, 2014, 2017) and time×groups (the 15 Fukushima municipalities, the nearby municipalities, the other municipalities) (*p* < 0.05; Table 3).

## 5. Discussion

In this study, among the places affected by the 2011 Great East Japan Earthquake and tsunami, we focused on 15 municipalities in the Fukushima Prefecture that were particularly devastated in 2011, and tested the medium-term effects (six years) of the major disaster and the tsunami on the long-term care certification rate and expenditure for provision of long-term care services.

### 5.1. Long-Term Care Certification Rate Spiked in Disaster Areas and Remained Higher Six Years Later

**Figure 1:**
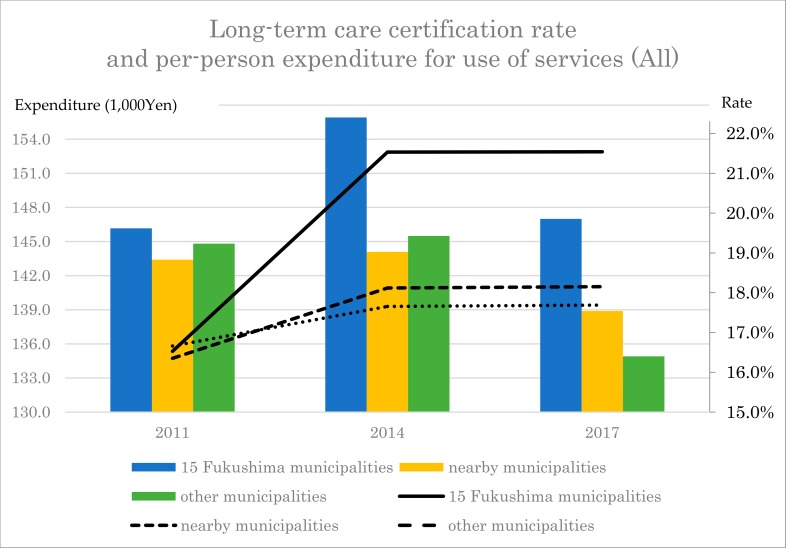
The change of long-term care certification rate and per-person expenditure for use of services for 6 years.

RM-ANOVA revealed the long-term care certification rate to be roughly the same or lower in the 15 Fukushima municipalities compared to the nearby municipalities and other municipalities in 2011, but 4.4% to 5.4% higher compared with the nearby and other municipalities after the earthquake, remaining 5.9% to 6.1% higher in 2017, six years after the earthquake. The per-person expenditure for use of services over one month did not differ among the three groups in 2011, but was higher in the 15 Fukushima municipalities (155,900 yen) compared to the nearby municipalities (143,100 yen) and other municipalities (145,500 yen) in 2014, with a similar trend observed in 2017 (Figure 1). The higher expenditure in 15 Fukushima municipalities can be due to the growth of the number of people with medium-level certification for long-term care, and due to the declining availability of a family caregiver than in the other two groups. 

**Figure 2:**
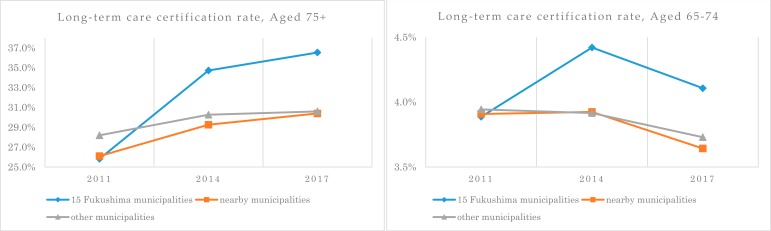
The change of long-term care certification rate for 6 years by age group.

In particular, the long-term care certification rate of the old-old in the 15 Fukushima municipalities was 25.9% in 2011, 34.7% in 2014, and 36.5% in 2017, rising 10.7% higher six years after the earthquake and 6.4% to 8.3% higher than the nearby municipalities (4.3% increase) and other municipalities (2.4% increase) (Figure 2). We did statistical comparisons that were stratified by long-term care level; however, we did not find unconventional views.

Dividing by type of need revealed that the ratio of those with mild or medium need increased more after the earthquake in the 15 Fukushima municipalities compared to the nearby municipalities and other municipalities in both the young-old and the old-old. From 2011 to 2014, the increase was especially pronounced among the old-old, who showed a 31.7% increase in the ratio of those with mild need (9.9% increase for nearby municipalities and 9.1% increase for other municipalities) and a 30% increase in the ratio of those with medium need (13.5% increase for nearby municipalities and 15.4% increase for other municipalities). The increase in the 15 Fukushima municipalities from 2014 to 2017 was roughly equal to that of the other two groups. For all aged 65 or over, a sharp increase in the long-term care certification rate in the 15 Fukushima municipalities has disappeared since 2014. The baby boomers after World War Ⅱ (born between 1947 and 1949) in Japan, which have a particularly large population by age group, have been included in the aged 65–74 since 2014. The proportion of young-old people (aged 65–74) with lower long-term care risk increased, while the proportion of old-old (aged 75 or older) with high long-term care risk decreased. Therefore, the long-term care certification rate of 2011–2014 has increased significantly, but the rate of 2014–2017 has not increased as much, partly because of stability after the disaster and change in the population structure. Nevertheless, the long-term care certification rate in the 15 Fukushima municipalities is higher than that of the other two groups even after six years since the earthquake. In particular, the certification rate and the long-term care certification for the old-old remain higher than the other two groups. The phenomenon that the certification rate increases after the earthquake might continue in the medium term.

In contrast, the ratio of those with major need decreased or only increased a small amount. The reason for this might be that the in-patient long-term care facilities used by many people with major care needs were damaged by the tsunami and staff at the facilities were affected by the tsunami [19,20], so the facilities for those who needed major care could not operate normally and those individuals were admitted to hospitals or long-term care facilities in areas not destroyed by the tsunami.

### 5.2. Factors Causing Increase in Long-Term Care Certification Rate and Countermeasures

The increase in those with mild or medium needs among those aged 75 years or older was dramatic in disaster-affected areas after the earthquake, and various studies have been conducted to examine the health impacts on people resulting from this earthquake.

A systematic review revealed psychological burdens such as a deterioration in mental health [21] and an increase in prevalence of post-traumatic stress disorder (PTSD) [22], as well as deterioration in health due to living in an evacuation center or a worse living environment [23], an increase in frailty risk [24], spread of pneumonia [25], and an increase in the number of people with subjective symptoms such as gastrointestinal and/or musculoskeletal symptoms [26].

Although a positive correlation was observed between the provision of medical and nursing care services and the long-term care certification rate [27], 10% of medical institutions were destroyed or closed due to the earthquake in disaster-affected areas [28], and the number of people at the hospitals decreased by 25.1% [29]. As the nursing care facilities themselves also sustained structural damage [19,20], it is unlikely that the increase in the long-term care certification rate was due to an increase in the amount of services provided.

Disaster survivors, and especially those aged 75 years or older, sustained not only direct injuries from the earthquake, but also deterioration in their living environments and psychological effects such as PTSD, as mentioned earlier. Informal care from family and others also declined [23], resulting in activity limitations. Together, these may have led to an increase in long-term care requirements for those with minor and medium need. This situation continued for a medium- to long-term duration. In other words, when a major disaster strikes, deterioration in the environment may lead to the need for long-term care. This suggests that strong and ongoing governmental support is needed to provide assistance with the tasks of daily living and promote social participation [30] to prevent those aged 75 years or older, who are particularly vulnerable to functional disability from losing physical and/or cognitive function, from requiring long-term care.

### 5.3. Study Limitations and Future Challenges

This study is one of the few studies that used national data from throughout Japan. However, the data used in this study do not provide any information on how individuals who were newly certified for long-term care after the earthquake were affected by the disaster, or their environment following the earthquake. The unreleased health data from Fukushima were a further limitation in this study. Further studies are needed to clarify the factors linked with functional decline and analyze the duration of the effects of a major disaster.

## 6. Conclusions

In this study, among the places affected by the 2011 Tohoku earthquake and tsunami, we focused on 15 coastal municipalities in the Fukushima Prefecture and analyzed the changes of the effects of a major disaster and tsunami on the long-term care certification rate and expenditure for provision of services over six years after the occurrence.

In 2011, we found no intergroup differences among the 15 Fukushima, nearby, and other municipalities in either the long-term care certification rate or per-person expenditure for use of services. In 2014, after the earthquake, the long-term care certification rate was 6.6% to 8.1% higher in the 15 Fukushima municipalities than in the nearby municipalities and other municipalities for those aged 75 years or older. Similarly, monthly per-person expenditure for use of services was 9900 to 11,800 yen higher in the 15 Fukushima municipalities than in the nearby and other municipalities in 2014, and this trend continued into 2017.

Previous studies indicated that people became certified for long-term care not only due to direct injuries in the earthquake, but also due to deterioration in their living environment after the earthquake and post-traumatic stress, and the adverse effects may be particularly notable in those aged 75 years or older, leading to mild or medium need for long-term care. Strong, ongoing governmental support is needed to assist older people, especially those aged 75 years or older, following a disaster.

## Figures and Tables

**Table 1:** Number of people eligible for long-term care and number of people receiving services.

				15 Fukushima Municipalities (*n* = 15)			Nearby Municipalities (*n* = 103)			Other Municipalities (*n* = 1460)		
				2011	2014	2017	2011	2014	2017	2011	2014	2017
Number of people certified for long-term care	Minor need	All	No. people	9379	12,283	13,437	87,798	106,002	117,273	2,073,450	2,531,699	2,827,512
			Rate of increase		31.0%	9.4%		20.7%	10.6%		22.1%	11.7%
		Aged 65–74	No. people	1104	1384	1478	10,985	12,434	13,225	290,945	346,169	367,262
			Rate of increase		25.4%	6.8%		13.2%	6.4%		19.0%	6.1%
		Aged 75+	No. people	8275	10,899	11,959	76,813	93,568	104,048	1,782,505	2,185,530	2,460,250
			Rate of increase		31.7%	9.7%		21.8%	11.2%		22.6%	12.6%
	Medium need	All	No. people	8410	10,746	11,696	64,677	74,352	79,502	1,445,858	1,640,433	1,785,245
			Rate of increase		27.8%	8.8%		15.0%	6.9%		13.5%	8.8%
		Aged 65–74	No. people	1051	1182	1284	7630	7912	8133	194,663	206,530	213,040
			Rate of increase		12.5%	8.6%		3.7%	2.8%		6.1%	3.2%
		Aged 75+	No. people	7359	9564	10,412	57,047	66,440	71,369	1,251,195	1,433,903	1,572,205
			Rate of increase		30.0%	8.9%		16.5%	7.4%		14.6%	9.6%
	Major need	All	No. people	7717	7997	7829	53,191	57,191	59,036	1,112,758	1,207,547	1,257,004
			Rate of increase		3.6%	−2.1%		7.5%	3.2%		8.5%	4.1%
		Aged 65–74	No. people	854	763	800	5771	5580	5815	131,903	137,186	138,034
			Rate of increase		−10.7%	4.8%		−3.3%	4.2%		4.0%	0.6%
		Aged 75+	No. people	6863	7234	7029	48,197	51,611	53,221	980,800	1,070,361	1,118,970
			Rate of increase		5.4%	−2.8%		7.1%	3.1%		9.1%	4.5%
Number of people receiving services	Minor need		No. people	7013	8735	10,441	64,333	76,203	87,489	1,510,199	1,831,646	2,127,178
			Rate of increase		24.6%	19.5%		18.5%	14.8%		21.3%	16.1%
	Medium need		No. people	8017	10,018	12,283	60,420	70,905	83,985	1,362,305	1,576,896	1,906,050
			Rate of increase		25.0%	22.6%		17.4%	18.4%		15.8%	20.9%
	Major need		No. people	7242	7441	7847	49,352	54,105	58,922	1,005,550	1,134,855	1,247,783
			Rate of increase		2.7%	5.5%		9.6%	8.9%		12.9%	10.0%
Aging rate			% (range)	26.5 (18.9–34.7)	28.4 (21.0–35.7)	31.3 (23.8–39.2)	28.1 (13.2–53.7)	29.6 (15.4–55.8)	32.3 (18.0–58.2)	27.4 (1.5–56.7)	29.4 (13.0–57.8)	32.0 (15.0–60.6)
Ratio of people aged 75 or older *			% (range)	57.6 (51.4–65.9)	56.5 (51.1–65.5)	53.5 (48.2–60.0)	56.2 (37.6–70.6)	56.2 (37.9–72.6)	54.2 (38.7–69.2)	52.5 (32.5–74.3)	52.1 (33.8–74.6)	51.8 (35.6–74.3)

* Ratio of people aged 75 years or older = over-75 population/over-65 population.

**Table 2:** Long-term care certification rate and per-person expenditure for use of services (analysis of RM-ANOVA).

Item		Year	15 Fukushima Municipalities (*n* = 15)	Nearby Municipalities (*n* = 103)	Other Municipalities (*n* = 1460)	Time	Time ×Group
			Mean	Mean	Mean	F	F
Long-Term Care Certification Rate	All ※	2011	16.5%	16.4%	16.7%	24.0 **	40.13 **
		2014	21.5%	18.1%	18.2%		
		2017	21.5%	17.7%	17.7%		
	Aged 65–74	2011	3.9%	3.9%	3.9%	2.90 **	1.10 **
		2014	4.4%	3.9%	3.9%		
		2017	4.1%	3.6%	3.7%		
	Aged 75+	2011	25.8%	26.1%	28.2%	377.8 **	62.8 **
		2014	34.7%	29.3%	30.3%		
		2017	36.5%	30.4%	30.6%		
Per-Person Expenditure for Use of Services※ (1000 yen)		2011	146.2	143.4	144.8	48.9 **	7.7 **
		2014	155.9	144.1	145.5		
		2017	147.0	138.9	134.9		

※ Covariates were assessed based on the value for the ratio of people aged 75 years or older for each year. Long-term care certification rate = Number of people certified for long-term care/Number of people aged 65 or older. ** *p* < 0.001.

**Table 3:** The result of post hoc test of RM-ANOVA.

		Time		Mean Difference	95% Confidence Interval	
		(I)	(J)	(I−J)	Lower	Upper
Long−Term Care Certification Rate	All	2011	2014 *	−0.025	−0.028	−0.022
			2017 *	−0.026	−0.030	−0.022
		2014	2011 *	0.025	0.022	0.028
			2017 *	−0.001	−0.004	0.002
		2017	2011 *	0.026	0.022	0.030
			2014 *	0.001	−0.002	0.004
	Aged 65−74	2011	2014 *	0.001	0.000	0.002
			2017 *	0.001	0.001	0.002
		2014	2011 *	−0.001	−0.002	0.000
			2017	0.000	0.000	0.001
		2017	2011 *	−0.001	−0.002	−0.001
			2014	0.000	−0.001	0.000
	Aged 75+	2011	2014 *	−0.002	−0.005	0.000
			2017 *	−0.004	−0.007	−0.001
		2014	2011 *	0.002	0.000	0.005
			2017	−0.001	−0.003	0.001
		2017	2011 *	0.004	0.001	0.007
			2014	0.001	−0.001	0.003
Per-Person Expenditure for Use of Services (1000 yen)		2011	2014 *	−3.346	−5.427	−1.266
			2017 *	5.411	2.523	8.299
		2014	2011 *	3.346	1.266	5.427
			2017 *	8.757	6.258	11.256
		2017	2011 *	−5.411	−8.299	−2.523
			2014 *	−8.757	−11.256	−6.258

* *p* < 0.05.
